# Biopsychosocial experiences and coping strategies of elderly ESRD patients: a qualitative study to inform the development of more holistic and person-centred health services in Singapore

**DOI:** 10.1186/s12889-019-7433-6

**Published:** 2019-08-14

**Authors:** Emeline Han, Farah Shiraz, Victoria Haldane, Joel Jun Kai Koh, Rina Yu Chin Quek, Semra Ozdemir, Eric Andrew Finkelstein, Tazeen Hasan Jafar, Hui-Lin Choong, Sheryl Gan, Lydia W. W. Lim, Helena Legido-Quigley

**Affiliations:** 1 Saw Swee Hock School of Public Health, National University of Singapore, 12 Science Drive 2, #10-03H, Tahir Foundation Building, Singapore, 117549 Singapore; 2 Duke NUS Medical School, 8 College Road, Singapore, 169857 Singapore; 3 Department of Renal Medicine, Singapore General Hospital, Outram Road, Singapore, 169608 Singapore

**Keywords:** End stage renal disease, ESRD, Elderly, Quality of life, Lived experiences, Coping mechanisms, Qualitative research

## Abstract

**Background:**

As the incidence and prevalence rates of end stage renal disease (ESRD) rise globally, a disproportionate increase has been observed in the elderly population. Singapore has the fifth highest incidence of treated ESRD worldwide, with the upward trend of ESRD being most apparent among those aged 70 years and older. Although it is well-documented that ESRD patients suffer an impaired quality of life compared to the general population, there is limited research focusing on the unique experiences and needs of elderly ESRD patients in Asian populations. To address the knowledge gap, this study seeks to explore the impact of ESRD and dialysis on the quality of life of elderly (≥70 years old) ESRD patients in Singapore and examine the coping strategies utilised by these patients.

**Methods:**

This qualitative study involved semi-structured, in-depth interviews with 7 peritoneal dialysis patients, 5 haemodialysis patients, 4 patients on non-dialysis supportive care and 7 caregivers in Singapore. Interviews were conducted in English, Chinese, and Malay and fully transcribed. QSR NVivo 11 software was used for analysis.

**Results:**

Participants reported that ESRD and dialysis had an impact on three highly interconnected areas of their quality of life: (a) biological/physical (general symptoms, neuromuscular problems, skin problems and poor sleep quality); (b) psychological (depressive symptoms, anxiety and fears, stress and negative self-perceptions); and (c) social (increased dependence on family and loss of social life). There were four key strategies that participants used to cope with these biopsychosocial challenges: (a) family support (financial, practical and emotional support); (b) religious/spiritual support (experiencing gratitude/contentment, the power of prayer and belonging to a faith community); (c) avoidance (cognitive avoidance and distraction techniques); and (d) acceptance (positive thinking and problem solving).

**Conclusions:**

This study has provided insights into the biopsychosocial impact of ESRD and dialysis, as well as cultural and religious factors that shape the experiences and coping mechanisms of elderly ESRD patients and caregivers in Singapore, which can be used to further the development and implementation of more holistic and person-centred services to help each patient achieve a better quality of life.

**Electronic supplementary material:**

The online version of this article (10.1186/s12889-019-7433-6) contains supplementary material, which is available to authorized users.

## Background

Chronic kidney disease is characterised by the progressive and irreversible loss of kidney function in removing toxins and excess fluids from the blood. The disease may be asymptomatic in its early stages, but as the disease progresses, its symptoms become increasingly debilitating and even fatal. In end stage renal disease (ESRD), the fifth and final stage of chronic kidney disease, common symptoms include dyspnoea, nausea, pain, fatigue, sleep disturbance, anxiety and depression [1]. The two main treatments for ESRD, haemodialysis (HD) and peritoneal dialysis (PD), are also associated with additional physiological and psychosocial stressors [2, 3]. The multi-faceted challenges of living with the illness and treatment, as well as the strategies that patients use to cope with them, have implications for various aspects of their quality of life [4–6].

Quality of life has been defined by the World Health Organisation (WHO) as “an individual’s perception of their position in life in the context of the culture and value systems in which they live and in relation to their goals, expectations, standards and concerns. It is a broad ranging concept affected in a complex way by the person’s physical health, psychological state, personal beliefs, social relationships and their relationship to salient features of their environment.” [7] There is evidence to suggest that ESRD patients suffer impaired quality of life compared to the general population and patients with some other chronic illnesses [8].

As the incidence and prevalence rates of ESRD rise globally, a disproportionate increase has been observed in the elderly population [9, 10]. However, there is limited research focusing on elderly ESRD patients and the available literature mainly addresses Western populations. Cross-cultural research has suggested that conceptualisations of wellbeing may differ between Asian and Western populations due to different constructions of selfhood. Markus and Kitayama explain that Western perceptions of the “self” focus on the individual’s unique traits, values and emotions that contribute towards autonomy and differentiation from others, while Asian perceptions of the “self” are concerned with the individual in relation to and interdependent on others [11, 12]. A qualitative study of haemodialysis patients in Hong Kong also showed that patients’ coping mechanisms were shaped by traditional Chinese philosophies, which have a different approach from Western philosophies to managing life stressors [13].

Singapore, a small but densely populated and rapidly ageing country in Southeast Asia, had the world’s fifth highest incidence of treated ESRD in 2016 [14]. According to the Singapore Renal Registry, the upward trend of ESRD is most apparent among those aged 70 years and older, and there is also a growing proportion of dialysis patients in this age group [15]. Studies in other countries have suggested that dialysis may confer minimal benefits in terms of survival and quality of life for ESRD patients above 75 years old with co-morbidities in comparison to conservative management, which focuses on symptom control and quality of life [16–18]. Thus, it is important to understand the impact of ESRD and dialysis on the quality of life of ESRD patients aged 70 and above in Singapore in order to provide targeted services and support for this population group. A qualitative study investigating the experiences and needs of HD patients aged 39–63 years in Singapore reported that main themes included emotional distress, treatment-related concerns and social support [19]. However, no study to date has examined the experiences and coping strategies of elderly (≥70 years old) ESRD patients in Singapore, whose needs may differ. To address this knowledge gap, this paper seeks to:

1. Explore the experience of elderly (≥70 years old) ESRD patients receiving HD, PD or non-dialysis supportive care in Singapore and its impact on their quality of life.
2. Examine the coping strategies utilised by elderly ESRD patients in Singapore.

## Methods

### Sampling

Purposive sampling was used to recruit ESRD patients who were receiving PD, HD or non-dialysis supportive care, and snowball sampling was used to recruit their caregivers (see Table 1 for complete inclusion criteria). Participants were recruited during appointments at Singapore General Hospital, the largest tertiary hospital in the country, with the assistance of renal coordinators, nurses and doctors.

**Table 1:** Inclusion Criteria

Patient	Caregiver
● Incident CKD Stage 5 with eGFR < 10 ml/min ● Aged 70 years or older ● Currently receiving haemodialysis OR peritoneal dialysis OR non-dialysis supportive care	● Primary informal caregiver of the patient ● Aged 21 years or older

### Data collection

In total, 62 participants were approached; 38 declined or were non-communicative, and 1 dropped out due to hospitalisation. Semi-structured, in-depth interviews were conducted with 23 participants from 4 participant categories: 7 PD patients, 5 HD patients, 4 patients on non-dialysis supportive care and 7 caregivers (Table 2). Interviews were conducted in English, Chinese, or Malay by researchers fluent in that language. Interviewers followed an interview guide (see Additional file 1) while conducting interviews. The guide included topics such as socio-demographics and caregiver support, medical history and medication, patient-provider relationships, decision-making processes and lived experiences. Data collection ceased when all researchers agreed that thematic saturation had been reached.

**Table 2:** Participant Characteristics Table (*N* = 23)

Patient Characteristics			
Characteristic	Female	Male	Total
Gender	9	7	16
Age Range			
71–75	3	6	9
76–80	5	1	6
81–85	1	0	1
Ethnicity			
Chinese	7	7	14
Malay	1	0	1
Indian	1	0	1
Dialysis Type			
Peritoneal Dialysis	3	4	7
Haemodialysis	4	1	5
Non-dialysis supportive care	2	2	4
Caregiver Characteristics			
Characteristic	Female	Male	Total
Gender	5	2	7
Ethnicity			
Chinese	2	4	6
Malay	1	0	1
Relationship to Patient			
Spouse	2	1	3
Child	1	1	2
Godchild	2	0	2

### Ethics

Ethical approval for the study was obtained from the SingHealth Centralised Institutional Review Board (CIRB). Informed consent for participation, audio recording and publication was obtained from all participants via a Participation Information Sheet and Consent Form. Interviews were conducted in private, quiet places to ensure participant comfort and confidentiality, and participants could discontinue their participation in the research at any time. Each excerpt in this paper includes the number of the interview and code letters (F for Female, M for male) so that extracts from the same individual can be linked. To maintain confidentiality, all names reported are pseudonyms and identifying data has been excluded.

### Data analysis

**Fig. 1:**
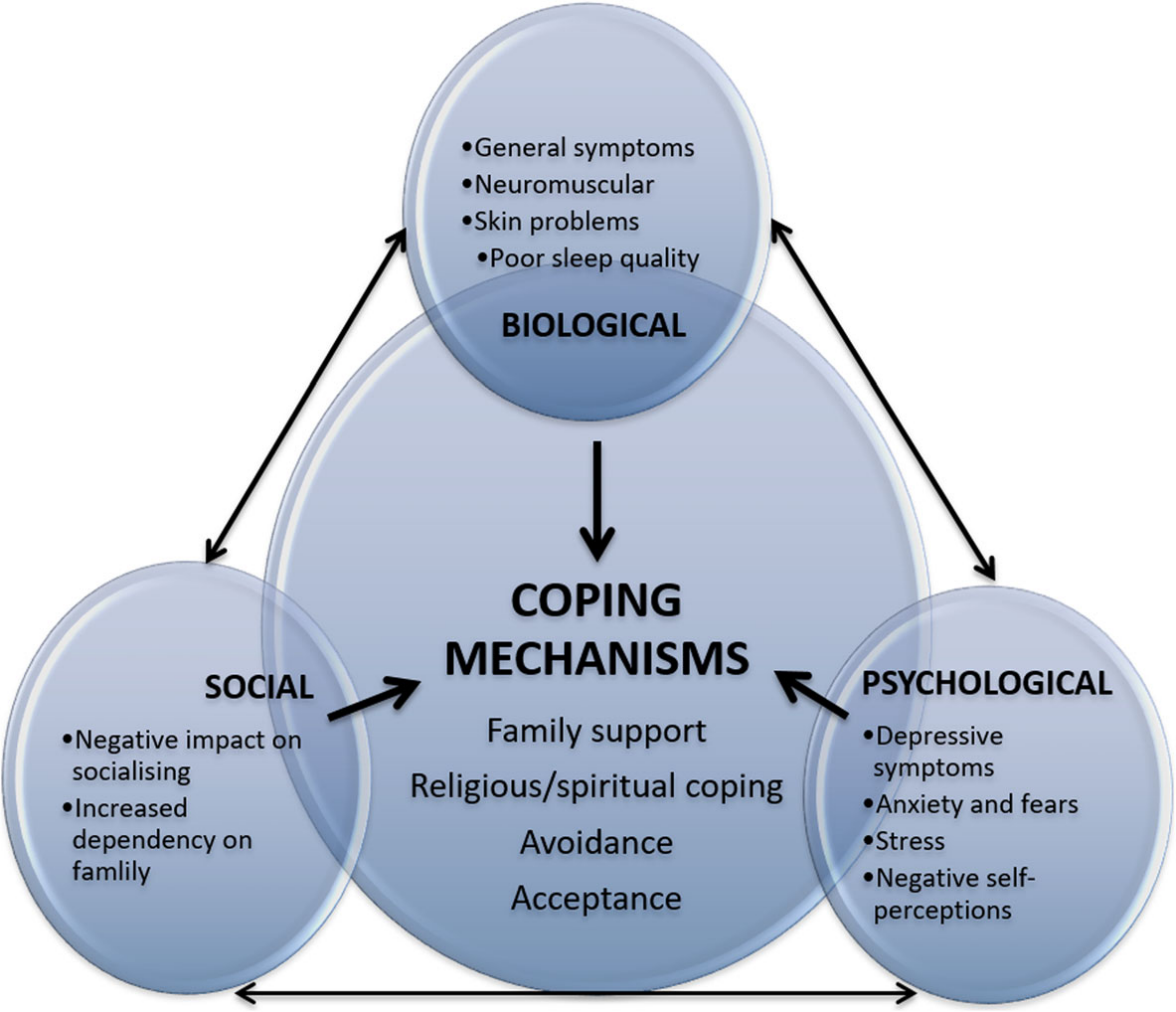
Conceptual framework of the biopsychosocial impact of ESRD and coping mechanisms of elderly ESRD patients

Interviews were recorded and then translated into English (if necessary) and transcribed in full. Two researchers trained in qualitative research methods coded interviews using QSR Nvivo 11 Software and held regular discussions to resolve discrepancies and reach a consensus on the final codes (see Additional file 2 for the Consolidated Criteria for Reporting Qualitative Studies). A combination of inductive and deductive strategies from framework analysis was used, including the five steps of familiarisation, identifying a thematic framework, indexing, charting and mapping, as well as interpretation [20]. As a main aim of this paper is to explore the impact of ESRD on quality of life within the sociocultural context of Singapore, the WHO’s cross-cultural model of quality of life was used to guide analysis, which encompasses six domains: physical, psychological, level of independence, social relations, environment and spirituality/religion [7]. Emergent themes were then used to inform the structure and content of our conceptual framework (Fig. 1).

## Results

Figure 1 illustrates the interconnectedness of the (a) biological/physical, (b) psychological, and (c) social impact of the ESRD diagnosis and treatment on quality of life as described by our participants. It also demonstrates the interconnectedness of these biopsychosocial challenges and the four main coping mechanisms used by participants, which were (a) family support, (b) religious/spiritual coping, (c) avoidance, and (d) acceptance.

We will describe the main themes and subthemes highlighted by participants on their experience of ESRD and dialysis and its impact on their quality of life, followed by the coping strategies utilised by these patients. A table of all the themes, sub themes and examples of evidence can be found in Additional file 3.

### Biopsychosocial experiences of ESRD and dialysis

Participants reported that ESRD and dialysis had an impact on three main areas of their quality of life: (a) biological/physical, (b) psychological, and (c) social (Fig. 1).

- **Biological/physical impact of ESRD and dialysis**

Participants described four key themes that had a significant impact on their physical health and which substantially affected their activities of daily living and quality of life. These included: (i) general symptoms such as dyspnoea, dizziness, oedema, nausea and loss of appetite, (ii) neuromuscular symptoms such as muscular aches and weakness causing mobility difficulties, (iii) skin problems such as itchy/dry skin, and (iv) poor sleep quality (Table 3). Poor sleep quality dominated the narratives of participants, where they described sleep-related problems such as difficulties falling asleep and frequent awakening which resulted in daytime sleepiness. For example, a PD patient reported sleep disturbances caused by the dialysis machine while undergoing dialysis overnight:

*“So the machine wakes you up every two or three hours, I’m okay, but still it’s quite stressful. You don’t get good quality sleep so the next day you feel still very drowsy.”*

*[PC11_Leong_M_PD]*

**Table 3:** Biological/physical impact of ESRD and dialysis

Theme	Subtheme	Verbatim extracts
General symptoms	Dyspnoea	“When I was sitting upright, I was fine. But when I was sleeping, I had trouble breathing.” [PB31_Huang_M _HD]
	Dizziness	“His blood pressure will be very low after dialysis, at around 80. He’ll feel dizzy and weak.” [C1PB31_Alice_F_HD_Godchild]
	Oedema	“I was all swollen, my leg, my hand, even my feet, all swollen with water. What to do? So swollen I can’t walk, so have to go for dialysis, that’s how I started.” [PB11_Siew Leng_F_HD]
	Nausea and appetite loss	“Keep on diarrhoea, vomiting and swelling everywhere...so warded GH here, then, at last, I can’t eat.” [PB11_Siew Leng_F_HD]
Neuromuscular symptoms	Muscular aches	“I went for dialysis this morning and my hip is aching now. I can’t walk.” [PB09_Ai Jia_F_HD]
	Muscular weakness	“I feel healthy but not so strong... if walking I must hold on something otherwise I feel tottery.” [PC10_Nur_F_PD]
Skin problems	Itchy/dry skin	“Then, the one time when I was dialysis on this ah, the time my body was very itchy...I hardly can sleep...that’s why my doctor prescribe this medication for me give me some drowsiness, then I have a good night sleep.” [PC16_Larry_M_PD]
Poor sleep quality	Physical causes	“So the machine wakes you up every two or three hours, I’m okay, but still it’s quite stressful. You don’t get good quality sleep so the next day you feel still very drowsy.” [PC11_Leong_M_PD]
	Psychological causes	“I couldn’t sleep for nights… Every night, I’d wake up every 2–3 h to think about it automatically. It’s a terrible thing… I was having sleepless nights thinking about it. It’s terrible as the same thing happened every day and every night; I couldn’t sleep.” [PA08_Bee Eng_F_ND]

Our findings revealed that poor sleep quality was not only due to physical factors such as ESRD symptoms and treatment, but also psychological factors such as significant worry following diagnosis:

*“I couldn’t sleep for nights… Every night, I’d wake up every 2-3 hours to think about it automatically. It’s a terrible thing… I was having sleepless nights thinking about it. It’s terrible as the same thing happened every day and every night; I couldn’t sleep.”*

*[PA08_Bee Eng_F _ND]*

(b) **Psychological impact of ESRD and dialysis**

Participants described experiencing elevated levels of psychological distress throughout various phases of the ESRD illness trajectory i.e. diagnosis, pre-treatment and post-treatment. The psychological impact of ESRD encompassed four key themes. Participants described suffering from (i) depressive symptoms, (ii) anxiety and fear, (iii) stress and (iv) negative perceptions of self. Each of these four themes incorporated several subthemes.

(i) **Depressive symptoms**

Depressive symptoms commonly reported by participants included low mood and sadness, as well as disengagement with others, following diagnosis of ESRD:

*“Yeah I see she is improving in appetite, she looks happier, uh, rather than before she looks moody, very sad, too much thinking, not talking.”*

*[CPA25_Cynthia_F_PD_Child]*

A few participants relayed thoughts of death and suicide. For example, difficulty accepting the initial diagnosis of such a serious illness led one participant to contemplate suicide:

*“Caregiver: She couldn’t accept it at first.”*

*“Patient: I wanted to commit suicide at first… I was never this sick and was so sad that I kept crying.”*

*[PB24_Mei Ling_F_HD]*

*[CPB24_Jun Hao_M_HD_Child]*

Another participant expressed thoughts of death underpinned by a sense of hopelessness and reluctance to be a burden on his family members:

*“Caregiver: His children saw how he was suffering; he could not sleep properly and had to keep sitting up. He told us to just let him die.”*

*“Interviewer: When the doctor first told you that you have to undergo dialysis, did he explain to you the various types of dialysis?”*

*“Patient: Yes. But I refused to do it. I’d rather give up and die.”*

*[PC42_Chia_M_PD]*

*[CPC42_Ying_F_PD_Spouse]*

(ii) **Anxiety and fear**

The anxiety and fears of participants stemmed from a fear of disease and treatment outcomes, including fear of the unknown, fear of pain and suffering, and fear of loss of freedom in daily life. One participant expressed her fear of injections and the life she associated with being on dialysis, including having to make lifestyle changes and losing the ability to engage in her hobbies (e.g. traveling), thereby taking away her enjoyment in life:

*“Interviewer: Were you scared of dialysis?”*

*“Interviewee: Scared of dialysis. That’s one thing.”*

*“Interviewer: Why?”*

*“Interviewee: Because I saw the equipment, I saw the needle and how your life is going to be you know. I mean I travel I enjoy travelling so I used to spend my time with my daughter. Everything will be gone. That’s the only pleasure I had.”*

*[PC32_Priya_F_PD]*

Fear of the unknown included uncertainty of treatment outcomes and lack of understanding of the treatment process due to insufficient information and preparation provided by healthcare professionals, which increased participants’ level of anxiety:

*“He… just oh you have to go for dialysis but never said what sort of dialysis…what will be the outcome…they took me to a room and that’s where I realized. They brought in one big needle ok…where are they going to insert this needle… Here comes the nurse they took my hand and they inserted the needle into the vein. Oh my god.”*

*[PC32_Priya_F_PD]*

In contrast, participants who felt well informed about their condition and details of treatment reported feeling less fearful of the disease and dialysis. A resourceful patient who independently sought out health information from books and the Internet explained:

***“*** *Who said you cannot sleep? It’s all in your mind; you are putting a lot of objection in front, obstacles. Why you’re putting obstacles, because of fear of the unknown. You know what is fear of unknown? You don’t know what to expect. But for me, once the unknown is known, the fear is no more.”*

*[PC11_Leong_M_PD]*

(iii) **Stress**

Participants identified stressors associated with living with ESRD such as the day-to-day stresses of managing their symptoms and treatment. In particular, PD patients reported additional stress due to the complexities of managing the dialysis process and equipment by themselves:

*“It’s a very stressful experience, but not a painful experience. What I mean by stressful is because the machine is set to first and foremost fill up, then after filling, it will dwell, which means it will stay in the peritoneal cavity for one and a half hour more or less, then after that they have to drain. It’s the draining process that’s very stressful. If your posture is not correct, an alarm will sound because not drawing enough fluid out. Then again, if we have constipation then also you’re in trouble. So there’s so many areas that you have to take care.”*

*[PC11_Leong_M_PD]*

(iv) **Negative self-perceptions**

Patients’ psychological distress was exacerbated by the perception of being a burden on others, especially their family members. Many participants experienced significant feelings of guilt and shame for the perceived financial and practical burden that they imposed on their children. For example, a PD patient perceived herself as “useless” and “a burden” because of the heavy expenses of dialysis that her daughter had to shoulder:

*“I mean you got children but you don’t want to be a burden to them also, for nothing you know for dialysis is running thousands of dollars for what. I mean inside me I really regret it’s a burden to my daughter but naturally, she won’t accept that. She says that’s a duty of a daughter to her mother you know according to her. However, deep inside me, I am useless and being a burden.”*

*[PC32_Priya_F_PD]*

On the other hand, a patient who refused to undergo dialysis explained his strong reluctance to impose a long-term caregiving responsibility on his children:

*“Think about it, if you have reached this age, even if you have children and grandchildren at home to take care of you, long-term care is a problem. Even if your son is capable, he has to take care of himself and his own family. Does he still have to take care of you? Can you bear to ask him to take care of you? If he starts taking care of you, he cannot stop taking care of you; but if he continues taking care of you, it’s a problem too… So when it’s time to go, we should just quickly go instead of being a burden to others. Even if we don’t hate chronic illnesses, other people will hate it.”*

*[PA19_Chiang Tee_M_ND]*

(c) **Social impact of ESRD and dialysis**

For many patients, ESRD symptoms and treatment led to an increased dependence on their family in day-to-day activities such as preparing meals, bathing and walking. Some patients also reported being dependent on family members to manage medication, wound care and dialysis. The impact of dialysis on family relationships was especially pronounced for PD patients, whose family members often had to adapt their daily schedules to help patients carry out dialysis at home. A spouse of a PD patient shared that language barriers hampered their ability to independently handle dialysis and medical appointments, resulting in lifestyle restrictions and inconveniences for their children:

*“It means our children also have no freedom. The instructions for the dialysis machine are all in English so we are afraid that we will use it wrongly. Once there’s a mistake, the machine’s alarm will go off. I’m a bit confused as I’m old already. So my children have no freedom. They can’t go out everyday; they must be at home to help him with dialysis. Every morning they need to attend to him before they can head to work. It is a little inconvenient for them. But, there is no choice since he has already reached this stage.”*

*[CPC42_Ying_F_PD_Spouse]*

Patients’ loss of independence in their daily life inevitably entailed a loss of social life as well. Some patients managed to maintain an active social life through engaging in group recreation activities (such as those at senior activity centres), church activities or even part-time jobs. However, those who experienced more severe physical symptoms and dependency often described a compromised social life characterised by being confined at home unless a family member was available to bring them out. For such patients, their social interaction was often limited to interaction with family:

*“Yeah usually be at home. Because, when she wants to go out she needs somebody to take her out with the wheelchair, cannot walk.”*

*[CPA25_Cynthia_F_PD_Child]*

Patients’ social lives were also adversely affected by the physically demanding and time consuming nature of dialysis. In particular, several HD patients shared that the frequent and lengthy dialysis sessions, traveling to the dialysis centre and post-dialysis fatigue left them with little or no time and energy to participate in social activities:

*“Tuesday, Thursday, Saturday, I have to get up very early to go there, then you can’t do much things these three days. Besides that you can’t go out or anything because you come back very tiring, that’s all.”*

*[PB11_Siew Leng_F_HD]*

### Coping Mechanisms and Strategies utilised by ESRD patients

There were four main strategies that participants used to cope with ESRD and dialysis: (a) family support, (b) religious/spiritual support, (c) avoidance, and (d) acceptance (Fig. 1).

- **Family Support**

In this study, family support stood out as an important tool in adapting to life with ESRD. It emerged as patients’ primary means of coping with the day-to-day challenges of the disease and treatment. Support from family commonly included financial, practical and emotional support:

*“Our children told him that they are willing to shoulder the burden and he needs to take care of himself… Fortunately, his children are all very filial… They are willing to put in the money and the effort. They also need to comfort him. To support him, everyone took leave to bring him to visit the doctor… There’s no choice at this stage. His children told him that money can be earned back and that he shouldn’t worry too much about it; they just need to work a bit harder and they can earn it back. But his heart aches for them.”*

*[CPC42_Ying_F_PD_Spouse]*

In particular, most patients sought financial support from their family members to cover their medical expenses, often as a first resort before turning to other sources. They perceived financial support from their family as a form of positive social support:

*“In the past, the doctor gave me this and said that if I have any difficulties, I can find the welfare organizations. I didn’t go for it. I can count on family members.”*

*[PC05_Seng_M_PD]*

At the same time, participants consistently reported feeling conflicted by receiving this form of support, expressing a reluctance to rely on others and a desire for independence. For example, several patients emphasised the importance of learning to self-manage their condition and treatment so as to minimise dependence on family members:

*“You know you have to learn. Even now I’m not very good at it in the night. On and off I am trying to…because I cannot carry the water bag, it’s very heavy. It’s quite a small distance only but I cannot. She’s very good at it so she’s training me.”*

*[PC32_Priya_F_PD]*

Nonetheless, most patients found themselves left with little choice but to rely on their families in some aspect or another due to their vulnerable circumstances. They acknowledged the need for interdependence in the ESRD journey, and the importance of involving their family members in each step, including treatment decision-making:

*“Interviewer: So the time that you make the decision to start your water dialysis, did you discuss with your family members and all that?”*

*“Patient: Of course. You can’t walk into that path alone. I do have a wife, I do have my children, and I can’t just walk in alone. If anything happens they’ll withdraw the support and I’m in trouble. So that cannot be happening.”*

*[PC11_Leong_M_PD]*

(b) **Religious/spiritual coping**

Religion/spirituality was another key coping strategy utilised by ESRD patients in this study. Religion imparted patients with a spiritual outlook on life and enabled them to face their illness with a resilient and optimistic attitude. A few patients reported counting their blessings, experiencing gratitude towards God and achieving a state of contentment with their lives:

*“Because I am so blessed, when I count over my blessings, I am more blessed than other people… I don't want to grumble… I have to be thankful and grateful for all that I have.”*

*[PA12_Irene_F_ND]*

A recurrent subtheme in participants’ responses was the power of prayer, which was often utilised as a method of coping with both the physical and emotional burden of ESRD. Patients described how surrendering their worries and problems to a higher power, as well as having faith and trust in God’s will, helped them to experience peace and hope:

*“If I have anything, so I leave it to God because I'm Christian…So they do pray for me. So I depend on divine will… I don't depend on my own strong will or fear, I only depend on God…Then God give me a message, that he will heal me. Then I got to be faithful and read his word and be close to God.”*

*[PA12_Irene_F_ND]*

Finally, participants reported belonging to a faith community as another valued aspect of religious coping, as they received financial support, practical support, emotional support and spiritual support (including prayer support) from fellow members of the community:

*“We go to XXX church. There are many believers and we are like family.”*

*[PB24_Mei Ling_F_HD]*

(c) **Avoidance**

Cognitive avoidance was commonly reported in participants living with ESRD. Most patients tried to avoid thinking about sickness and death, which for some included not wanting to know too much about their condition and treatment:

*“Interviewer: Before you started dialysis, what did you know about it?”*

*“Interviewee: I didn’t know much about it. There’s no choice since I’m sick so I don’t really think about it much. I try not to worry about it that much. It’s necessary to undergo dialysis since I’m sick. This sickness must be treated this way. No dialysis cannot, I try not to think too much about it.”*

*[PC05_Seng_M_PD]*

Some participants used distraction techniques such as engaging in work or hobbies, exercising, watching television and conversing with others about topics other than their disease. One participant, who runs a karaoke studio for elderly people, explained how this form of escapism helps to improve his mood:

*“At this age, I’d only be waiting for death at home. Why am I here when I don’t earn much money? It’s because I have food to eat and songs to sing. So I live day by day. I don’t think about dying… when I sing, I won’t think so much. Thinking too much will cause you to be in a bad mood and this will make it difficult for those around you to take care of you.”*

*[PA19_Chiang Tee_M_ND]*

(d) **Acceptance**

In contrast to the avoidant coping strategies reflected above, the two extracts below show how some patients accepted and adjusted to the changes in their lives brought about by ESRD. They utilised positive thinking and problem solving to mitigate the negative impact of the disease and treatment on their quality of life. One participant described his process of accepting dialysis and its impact on his daily life, which involved reframing negative thoughts and focusing on the positive:

*“You see, I avoided dialysis because I don’t want to change my daily life. Then when I take the next step, I have to accept what is coming. So it’s a matter of how I would psychologically influence myself. I think a lot of psychological effects -- I went in, okay, fair enough, besides 10 hours on the bed, in the day I come up and do a walk around, I feel very fresh.”*

*[PC11_Leong_M_PD]*

Problem-focused coping involved defining the problem and generating alternative solutions. One participant recounted how she embraced the loss of her previous hobbies and chose to make the best of her situation by finding new passions to pursue:

*“Yes I mean don’t put it in my mind, don’t take it into your mind, oh that I’m a dialysis patient you know… pity yourself and all that… so what will you do? You have to make the best of everything. Not say it’s the end of the world… It’s very boring. But no matter what, I’m finding for a part-time job to keep me going. I do volunteer work in the meantime I am doing. The other thing is, you must have heard about the allotment gardens the government. On top of it is, I’m very passionate about plants. I’m very passionate, so that’s the thing to keep me going. Because I am very passionate and I have a very positive thinking. It is what you know I’d have exercise and I will be losing weight it will be a regular thing.”*

*[PC32_Priya_F_PD]*

## Discussion

Our findings showed that ESRD and dialysis had a significant impact on patients’ quality of life across the physical, psychological, and social domains. In the physical domain, our findings were similar to a quantitative study in the Netherlands, which found that the three most common symptoms clusters identifiable in ESRD patients were general symptoms of the uraemic syndrome (e.g. dyspnoea, dizziness, nausea), neuromuscular problems (e.g. sore muscles) and skin problems (e.g. itchy/dry skin) [21]. In addition, a fourth symptom commonly reported by our participants was poor sleep quality, and our findings revealed that this was caused not just by physical factors but also psychological factors such as significant worry. This supports other studies reporting that poor sleep is prevalent in ESRD patients and is associated with depression [22–24], illustrating the relationship between physical and psychological wellbeing.

In the psychological domain, our participants described experiencing depressive symptoms, anxiety, fear, stress and negative self-perceptions, which were also linked to the impact of ESRD and dialysis on their personal relationships and social life (the social domain). Many participants spoke of how ESRD increased their level of dependence on family members and created a perception of being a burden on them, which resulted in significant feelings of guilt and shame. This contrasts with ESRD patients in a qualitative study in the United Kingdom who highlighted undermined body image, self-esteem and sexual activity/intimacy as significant psychosocial burdens [25]. These findings support cross-cultural research suggesting that self-oriented dimensions of wellbeing feature more prominently within Western populations while other-oriented dimensions of wellbeing feature more prominently in Asian populations [26].

The interconnectedness of the physical, psychological, and social impact of ESRD on quality of life underscores the need for a holistic and integrated approach to providing renal support services. However, the literature suggests that there is a tendency for renal service providers to focus on the physical wellbeing of ESRD patients and that mental health problems are underdiagnosed as well as undertreated in this population [27, 28]. A prevalence study in Singapore found that 49.9 and 45.5% of adult ESRD patients reported elevated depressive symptoms and anxiety symptoms respectively, and age was not a factor associated with either symptom [29]. This evidence and the presence of similar symptoms among our participants highlights the importance of a multidisciplinary ESRD care team that includes mental health professionals such as a psychiatric nurse, psychologist and/or psychiatrist [30]. Nurses and/or social workers should be trained to screen ESRD patients for anxiety and depression routinely and then refer high-risk patients to the appropriate specialists for formal diagnosis and treatment [31]. Additionally, educational initiatives addressing patients’ and caregivers’ abilities to recognise mental illnesses and knowledge of professional help available may help to reduce stigmatising attitudes and facilitate appropriate help-seeking among the Singapore population, which has reportedly poor mental health literacy [32]. A well-coordinated and comprehensive care system is needed to ensure timely intervention responding to the full range of needs of ESRD patients.

Our results also showed that regardless of the area of quality of life affected, elderly ESRD patients utilised four main coping mechanisms, namely family support, religious coping, avoidance, and acceptance. Family support was the most commonly used coping strategy among our participants. Patients reported seeking and receiving support from family members, especially their children, in the form of financial, practical and emotional support. Participants’ responses again reflected Asian values such as collectivism and filial piety, according to which family members have a responsibility to take care of each other, and children in particular are obligated to care for their parents/elders [33]. In Singapore, these values and expectations are embedded in the health system and policy structures that emphasise family as the first line of support before others and the government. For example, Medisave, a mandatory health savings scheme, allows immediate family members to use the money from each other’s saving accounts to pay for medical expenses [34]. Thus, it is unsurprising that most of our participants chose to seek financial support from their family, even if they felt apologetic for doing so, before turning to government welfare schemes. Studies have shown that stronger perceived family support is associated with reduced depression in ESRD patients [35, 36], but at the same time, if patients engage in self-blame because of the perceived burden on their family, this can also result in depression [37, 38].

The interconnectedness of patients’ and family members’ wellbeing, as well as patients’ heavy reliance on family support as a coping mechanism, also highlights the importance of extending renal support services to the family caregivers of elderly ESRD patients. A recent study in Singapore reported that family caregivers faced significant challenges in providing practical, emotional and financial support for patients with ESRD and dialysis, and that the caregiving role had a negative impact on physical and psychological well-being, as well as employment status [39]. The need to provide support for patients’ families/caregivers is often overlooked, and this need is magnified in Asian societies where families bear the primary responsibility of caring for patients, and where the wellbeing of family members are intimately intertwined with one another. In this context, it is clear that alleviating the burdens of family caregivers by facilitating their access to services providing both instrumental and emotional support will be crucial to improving the quality of life of elderly ESRD patients.

Religion/spirituality was another prominent coping strategy employed by participants in this study. These patients shared how their faith enabled them to remain thankful towards God despite their circumstances, experience relief from the burdens of ESRD through the power of prayer, as well as gain support from other members of their faith community. Indeed, several studies have shown that religion and spirituality correlate with better mental health, lower suicide risk, increased perception of social support and improved quality of life [40, 41]. However, in contrast to the positive religious coping exemplified by our participants, other studies have identified patients experiencing negative religious coping, also known as religious struggle [42, 43]. Religious struggle has been associated with greater psychological distress and poorer quality of life in patients with ESRD and other chronic illnesses [44, 45] as well as increased mortality among older patients [46].

The significant impact that religious coping methods have on patients’ quality of life provides a basis for incorporating religious/spiritual interventions in ESRD care aimed at promoting positive religious coping and minimising religious struggle [47]. However, religion and spirituality are often neglected in daily clinical practice, and healthcare professionals in Singapore as well as other countries have reported a lack of knowledge and training in these areas [48, 49]. The prominence of religion/spirituality as a coping mechanism of elderly ESRD patients in Singapore necessitates education and training for renal service providers, as well as collaboration between service providers and religious leaders, to adequately deal with religious and spiritual issues in ESRD as well as explore the possibility of integrating religion and spirituality into clinical practice [48].

Furthermore, there was a distinction between the patients in our sample who used avoidance-based versus acceptance-based coping strategies. In congruence with the findings of Gilbar et al. [50], those who showed more avoidance also tended to use more emotion-focused coping, while those who showed more acceptance tended to use more problem-focused coping. In the literature, emotion-focused coping has been associated with inferior outcomes in both the physical and psychological components of quality of life as compared to problem-focused coping [51–53]. In the same vein, avoidant coping has also been associated with worse mental health and increased risk for mortality [54–56]. However, several studies have clarified that it is behavioural avoidance (i.e. the failure to engage in problem-solving behaviours such as seeking medical help) that mediates the association between avoidant coping and poorer patient outcomes [55, 56], and that cognitive avoidance alone (such as using distraction techniques while still adhering to one’s treatment regimen) is not associated with negative outcomes and may even be beneficial for ESRD patients’ emotional status and quality of life [50, 56, 57].

Research has also found that ESRD patients’ treatment adherence and quality of life are improved when their coping styles are congruent with the demands of their treatment. For example, patients with more active coping styles show better adherence to peritoneal dialysis which is self-administered at home, while patients with more passive coping styles show better adherence to haemodialysis which is administered by staff at a dialysis centre or hospital [58]. This shows the importance of evaluating the coping styles of elderly ESRD patients in Singapore and providing person-centred services that take into account individual patients’ personalities, abilities, needs and resources. Ultimately, an individualised approach is required to help each ESRD patient arrive at an appropriate combination of problem-focused and emotion-focused strategies to cope with the whole spectrum of the ESRD burden and improve their quality of life.

### Strengths and limitations

This study is the first study to provide a unique insight into the lived experiences of elderly ESRD patients above the age of 70 and their caregivers within an Asian population from a biopsychosocial perspective. It has also highlighted the needs of these patients and caregivers and developed contextualised recommendations that can be used to shape and improve future services for similar population groups.

However, the presence of potential participant bias should be acknowledged as many patients approached had declined to take part in the study due to being too frail, non-communicative or unwilling to talk about their experience. This means that our findings may be reflective of a cohort of patients who are coping better with ESRD than those who are more vulnerable. In addition, although our study included caregivers, it focused more on patients’ wellbeing and family caregiving as a coping mechanism. Further research focusing on caregivers is required to examine the biopsychosocial impact of ESRD and dialysis on their quality of life. Moreover, the small sample size, paucity of non-Chinese participants and lack of data on each participant’s religion and length of stay in Singapore limit the generalisability of our findings. Future research should include a larger sample with more Malay and Indian participants as well as collect more relevant data on participants’ characteristics. Finally, although we observed some differences between our study population and Western study populations, we cannot conclude that these differences were due to culture-specific factors as we did not directly compare Asian and Western participants. A comparative study incorporating both Asian and Western participants would help to clarify the possible cultural differences.

## Conclusion

In cross-cultural settings, it is necessary for service providers to be aware of and accommodate patients’ differing views, values and preferences. This study has provided insights into the biopsychosocial impact of ESRD and dialysis, as well as cultural and religious factors that shape the experiences and coping mechanisms of elderly ESRD patients and caregivers in Singapore, which can be used to further the development and implementation of more holistic and person-centred services to help each patient achieve a better quality of life.

## Additional files

Additional file 1: Interview Guide. (DOCX 16 kb)

Additional file 2: Consolidated criteria for reporting qualitative studies (COREQ): 32-item checklist. (DOCX 20 kb)

Additional file 3: Table of key themes, subthemes and verbatim extracts examining the biopsychosocial impact of ESRD and coping mechanisms of elderly ESRD patients and caregivers (*n* = 23). (DOCX 22 kb)

## Data Availability

The datasets generated and/or analysed during the current study are not publicly available due to the confidential and sensitive nature of our data. As participant consent and ethical approval have not been obtained for data sharing, there is no data that can be disclosed beyond that contained within this paper.

## Abbreviations

ESRD: End Stage Renal Disease
HD: Haemodialysis
PD: Peritoneal Dialysis
WHO: World Health Organisation
