# Supplementary material for: Biopsychosocial experiences and coping strategies of elderly ESRD patients: a qualitative study to inform the development of more holistic and person-centred health services in Singapore
Source: BMC Public Health. 2019 Aug 14;19:1107. doi: 10.1186/s12889-019-7433-6 (PMC6694659; doi:10.1186/s12889-019-7433-6)
Supplement: Supplementary file 3 — Table of key themes, subthemes and verbatim extracts examining the biopsychosocial impact of ESRD and coping mechanisms of elderly ESRD patients and caregivers (n = 23). (DOCX 22 kb) [file 12889_2019_7433_MOESM3_ESM.docx]

**Additional File 3. Table of key themes, subthemes and verbatim extracts examining the biopsychosocial impact of ESRD and coping mechanisms of elderly ESRD patients and caregivers (n=23)**

| **Theme** | **Subtheme** | **Verbatim extracts** |
| --- | --- | --- |
| **Biological/Physical Impact of ESRD and Dialysis** | | |
| General symptoms | Dyspnoea | “When I was sitting upright, I was fine. But when I was sleeping, I had trouble breathing.” [PB31_Huang_M _HD] |
|  | Dizziness | “His blood pressure will be very low after dialysis, at around 80. He’ll feel dizzy and weak.” [C1PB31_Alice_F_HD_Godchild] |
|  | Oedema | “I was all swollen, my leg, my hand, even my feet, all swollen with water. What to do? So swollen I can’t walk, so have to go for dialysis, that’s how I started.” [PB11_Siew Leng_F_HD] |
|  | Nausea and appetite loss | “Keep on diarrhoea, vomiting and swelling everywhere...so warded GH here, then, at last, I can't eat.” [PB11_Siew Leng_F_HD] |
| Neuromuscular symptoms | Muscular aches | “I went for dialysis this morning and my hip is aching now. I can’t walk.” [PB09_Ai Jia_F_HD] |
|  | Muscular weakness | “I feel healthy but not so strong... if walking I must hold on something otherwise I feel tottery.” [PC10_Nur_F_PD] |
| Skin problems | Itchy/dry skin | “Then, the one time when I was dialysis on this ah, the time my body was very itchy...I hardly can sleep...that's why my doctor prescribe this medication for me give me some drowsiness, then I have a good night sleep.” [PC16_Larry_M_PD] |
| Poor sleep quality | Physical causes | “So the machine wakes you up every two or three hours, I’m okay, but still it’s quite stressful. You don’t get good quality sleep so the next day you feel still very drowsy.” [PC11_Leong_M_PD] |
|  | Psychological causes | “I couldn’t sleep for nights… Every night, I’d wake up every 2-3 hours to think about it automatically. It’s a terrible thing… I was having sleepless nights thinking about it. It’s terrible as the same thing happened every day and every night; I couldn’t sleep.” [PA08_Bee Eng_F_ND] |
| **Psychological Impact of ESRD and Dialysis** | | |
| Depressive symptoms | Low mood and sadness | “Yeah I see she is improving in appetite, she looks happier, uh, rather than before she looks moody, very sad, too much thinking, not talking.” [CPA25_Cynthia_F_PD_Child] |
|  | Disengagement with others | “He wasn’t like this before. He wouldn’t be sitting here talking to you like this. When we talked to him, he would just ignore us. When he couldn’t lie down because he couldn’t breathe properly, he didn’t tell us. His children asked him why he kept sleeping while sitting up, but he just said that he couldn’t sleep.” [CPC42_Ying_F_PD_Spouse] |
|  | Thoughts of death and suicide | “Caregiver: She couldn’t accept it at first.  Patient: I wanted to commit suicide at first… I was never this sick and was so sad that I kept crying.” [PB24_Mei Ling_F_HD] |
| Anxiety and fear | Fear of the unknown | “He… just oh you have to go for dialysis but never said what sort of dialysis…what will be the outcome…they took me to a room and that’s where I realized. They brought in one big needle ok…where are they going to insert this needle… Here comes the nurse they took my hand and they inserted the needle into the vein. Oh my god.” [PC32_Priya_F_PD] |
|  | Fear of pain and suffering | “I was only scared of the pain of injections but I’m not scared after that.” [PB09_Ai Jia_F_HD] |
|  | Fear of loss of freedom | “Because I saw the equipment, I saw the needle and how your life is going to be you know. I mean I travel I enjoy travelling so I used to spend my time with my daughter. Everything will be gone. That’s the only pleasure I had.” [PC32_Priya_F_PD] |
| Stress | Stress of managing symptoms and treatment | “It’s a very stressful experience, but not a painful experience. What I mean by stressful is because the machine is set to first and foremost fill up, then after filling, it will dwell, which means it will stay in the peritoneal cavity for one and a half hour more or less, then after that they have to drain. It’s the draining process that’s very stressful. If your posture is not correct, an alarm will sound because not drawing enough fluid out. Then again, if we have constipation then also you’re in trouble. So there’s so many areas that you have to take care.” [PC11_Leong_M_PD] |
| Negative self-perceptions | Perception of being a burden | “I mean you got children but you don’t want to be a burden to them also, for nothing you know for dialysis is running thousands of dollars for what. I mean inside me I really regret it’s a burden to my daughter but naturally, she won’t accept that. She says that’s a duty of a daughter to her mother you know according to her. However, deep inside me, I am useless and being a burden.” [PC32_Priya_F_PD] |
| **Social Impact of ESRD and Dialysis** | | |
| Impact on social life | Negative impact on socialising | “Tuesday, Thursday, Saturday, I have to get up very early to go there, then you can’t do much things these three days. Besides that you can’t go out or anything because you come back very tiring, that’s all.” [PB11_Siew Leng_F_HD] |
| Impact on personal relationships | Increased dependence on family | “It means our children also have no freedom. The instructions for the dialysis machine are all in English so we are afraid that we will use it wrongly. Once there’s a mistake, the machine’s alarm will go off. I’m a bit confused as I’m old already. So my children have no freedom. They can’t go out everyday; they must be at home to help him with dialysis. Every morning they need to attend to him before they can head to work. It is a little inconvenient for them. But, there is no choice since he has already reached this stage.” [CPC42_Ying_F_PD_Spouse] |
| **Coping Mechanisms** | | |
| Family support | Financial support | “There’s no choice at this stage. His children told him that money can be earned back and that he shouldn’t worry too much about it; they just need to work a bit harder and they can earn it back. But his heart aches for them.” [CPC42_Ying_F_PD_Spouse] |
|  | Practical support | Interviewer: Do you know what kind of medication are you eating?  Interviewee: No, I’m not sure. My daughter helps me to organise my medications into daily portions. [PA31_Wen Xi_M_ND] |
|  | Emotional support | “Our children told him that they are willing to shoulder the burden and he needs to take care of himself… Fortunately, his children are all very filial… They are willing to put in the money and the effort. They also need to comfort him. To support him, everyone took leave to bring him to visit the doctor…” [CPC42_Ying_F_PD_Spouse] |
| Religious/spiritual coping | Gratitude and contentment | “Because I am so blessed, when I count over my blessings, I am more blessed than other people… I don't want to grumble… I have to be thankful and grateful for all that I have.” [PA12_Irene_F_ND] |
|  | Power of prayer | “If I have anything, so I leave it to God because I'm Christian…So they do pray for me. So I depend on divine will… I don't depend on my own strong will or fear, I only depend on God…Then God give me a message, that he will heal me. Then I got to be faithful and read his word and be close to God.” [PA12_Irene_F_ND] |
|  | Faith community | “We go to XXX church. There are many believers and we are like family.” [PB24_Mei Ling_F_HD] |
| Avoidance | Cognitive avoidance | “Interviewer: Before you started dialysis, what did you know about it?  Interviewee: I didn’t know much about it. There’s no choice since I’m sick so I don’t really think about it much. I try not to worry about it that much. It’s necessary to undergo dialysis since I’m sick. This sickness must be treated this way. No dialysis cannot, I try not to think too much about it.”  [PC05_Seng_M_PD] |
|  | Distraction techniques | “At this age, I’d only be waiting for death at home. Why am I here when I don’t earn much money? It’s because I have food to eat and songs to sing. So I live day by day. I don’t think about dying… when I sing, I won’t think so much. Thinking too much will cause you to be in a bad mood and this will make it difficult for those around you to take care of you.” [PA19_Chiang Tee_M_ND] |
| Acceptance | Positive thinking | “You see, I avoided dialysis because I don’t want to change my daily life. Then when I take the next step, I have to accept what is coming. So it’s a matter of how I would psychologically influence myself. I think a lot of psychological effects -- I went in, okay, fair enough, besides 10 hours on the bed, in the day I come up and do a walk around, I feel very fresh.” [PC11_Leong_M_PD] |
|  | Problem solving | “Yes I mean don’t put it in my mind, don’t take it into your mind, oh that I’m a dialysis patient you know… pity yourself and all that… so what will you do? You have to make the best of everything. Not say it’s the end of the world… It’s very boring. But no matter what, I’m finding for a part-time job to keep me going. I do volunteer work in the meantime I am doing. The other thing is, you must have heard about the allotment gardens the government. On top of it is, I’m very passionate about plants. I’m very passionate, so that’s the thing to keep me going.” [PC32_Priya_F_PD] |
